# The Role of Citicoline in Neuroprotection and Neurorepair in Ischemic Stroke

**DOI:** 10.3390/brainsci3031395

**Published:** 2013-09-23

**Authors:** José Álvarez-Sabín, Gustavo C. Román

**Affiliations:** 1 Neurovascular Unit, Department of Neurology, Universitat Autónoma de Barcelona, 119-129 Passeig de la Vall d’Hebron, Barcelona 08035, Spain; E-Mail: josalvarez@vhebron.net; 2 Department of Neurology, Nantz National Alzheimer Center, Methodist Neurological Institute, Houston, TX 77030, USA

**Keywords:** citicoline, neuroprotection, neurorepair, ischemic stroke, hemorrhagic stroke, vascular dementia, vascular cognitive disorders, neurogenesis, synaptogenesis, neuronal plasticity

## Abstract

Advances in acute stroke therapy resulting from thrombolytic treatment, endovascular procedures, and stroke units have improved significantly stroke survival and prognosis; however, for the large majority of patients lacking access to advanced therapies stroke mortality and residual morbidity remain high and many patients become incapacitated by motor and cognitive deficits, with loss of independence in activities of daily living. Therefore, over the past several years, research has been directed to limit the brain lesions produced by acute ischemia (neuroprotection) and to increase the recovery, plasticity and neuroregenerative processes that complement rehabilitation and enhance the possibility of recovery and return to normal functions (neurorepair). Citicoline has therapeutic effects at several stages of the ischemic cascade in acute ischemic stroke and has demonstrated efficiency in a multiplicity of animal models of acute stroke. Long-term treatment with citicoline is safe and effective, improving post-stroke cognitive decline and enhancing patients’ functional recovery. Prolonged citicoline administration at optimal doses has been demonstrated to be remarkably well tolerated and to enhance endogenous mechanisms of neurogenesis and neurorepair contributing to physical therapy and rehabilitation.

## 1. Introduction

Each year, about 22 million people worldwide suffer a stroke. Stroke is a global health-care problem that causes a substantial burden of disease and remains one of the most devastating public health problems, often resulting in death or severe physical impairment and disability. According to the *Global Burden of Disease Study 2010* [1] in the last decade stroke became the third-most-common global cause of disability-adjusted life years (DALYs), second only to ischemic heart disease [1]. Increase in vascular risk factors—in particular, high blood pressure, tobacco smoking, alcohol, and poor diets—appears to be responsible for this increase [1]. Although effective primary prevention can be achieved with measures controlling vascular risk factors, at present, there are only two effective evidence-based treatments for stroke: stroke unit care and thrombolysis with alteplase (recombinant tissue plasminogen activator, rtPA).

Ischemic stroke is a dynamic process whereby the longer the arterial occlusion persists the larger the infarct size becomes and the higher the risk of post-perfusion hemorrhage. The goal of ischemic stroke treatment is to reopen the occluded artery. The only treatment that has demonstrably been able to halt the dynamic process launched by the vessel occlusion is rtPA that increases five times the odds of early recanalization (in the first 6 h), resulting in a decrease in infarct size with better neurologic and functional outcome of the patient [2]. Currently, intravenous fibrinolysis can be administered safely within the first 4.5 h following stroke onset [3]; and even as late as 6 h when an arterial occlusion is demonstrated with presence of potentially salvageable tissue (ischemic penumbra). In these late cases, the results of fibrinolysis treatment are similar to those of earlier windows in terms of arterial recanalization, functional recovery and frequency of hemorrhagic transformation [4].

Intravenous thrombolysis can be reinforced with ultrasound-enhanced treatment or sonothrombolysis [5] and ultrasound plus microbubbles [6]. Arterial recanalization in acute stroke can also been achieved by interventional neurovascular treatments including combined *i.v*. thrombolysis plus intra-arterial rescue in cases refractory to *i.v*. rtPA thrombolysis [7]. In acute stroke patients where rtPA is contraindicated other therapeutic options include primary intra-arterial thrombolysis and/or mechanical thrombectomy [8].

A recent systematic review and meta-analysis comparing intra-arterial thrombolysis *vs*. standard treatment or intravenous thrombolysis in adults with acute ischemic stroke demonstrated a modest benefit of intra-arterial thrombolysis over standard treatment, although no clear benefit was found for intra-arterial thrombolysis over intravenous thrombolysis in acute ischemic stroke patients [9]. However, there was an almost fourfold increase in risk of intracranial hemorrhage (RR = 3.90; 95% CI 1.41–10.76; *p* = 0.006) with intra-arterial thrombolysis [9]. In a study conducted by Álvarez-Sabin and colleagues [10], diffusion-weighted magnetic resonance imaging (DW-MRI) was performed in a group of patients with acute ischemic stroke involving the middle cerebral artery (MCA) territory. Initial DW-MRI was obtained within 6 h after ictus and was repeated 36–48 h later; images demonstrated increased size of the lesions in 77% of the patients. However, lesion-size increase was significantly smaller in those treated with *i.v*. rtPA than in those untreated (57.7% *vs*. 234.7%).

Therefore, recanalization treatment only controls partially the biochemical and molecular events triggered by cerebral ischemia, indicating that other factors must be controlled [11,12]; such factors include, but are not limited to, collateral blood flow, body temperature, hyperglycemia [13,14,15,16], and blood pressure fluctuations. Ideally, sufficient protection must be provided to the ischemic brain (neuroprotection) along with enhanced recovery of the damaged brain (neurorepair).

Finally, incorporating stroke unit care and thrombolysis into medical services is difficult and even impossible in many low- and middle-income countries—which have the greatest burden of stroke—because the required high levels of infrastructure, expertise, and resources are unavailable. Therefore, safe and effective neuroprotective drugs that could be given at medical services with limited resources would improve the outcome of millions of acute stroke patients.

## 2. Ischemic Neuroprotection: Brain Protection

Ischemic neuroprotection (brain protection) may be defined as any strategy, or combination of strategies, that antagonizes, interrupts, or slows down the sequence of injurious biochemical and molecular events that, if left unchecked, eventually result in irreversible ischemic injury [12]. Neuroprotection attempts to limit the brain damage produced by ischemia.

Experimental studies have demonstrated the complexity of the pathophysiology of stroke [17,18,19,20]. Among others, it involves excitotoxicity mechanisms [18], oxidative stress damage [19,20,21], inflammatory pathways [22,23], ionic imbalances, apoptosis, and angiogenesis [24,25] that are potential targets being evaluated in clinical trials [17,18]. Although successful in experimental models, translation to bedside treatments has been disappointing and complicated by some of the following reasons:

1. There is a need to protect the entire neurovascular unit that comprises neurons, glia, pericytes and blood vessels [26,27]. For many years the goal was to salvage neurons in the ischemic penumbra but recently it became clear that this goal is insufficient and that all the elements of the neurovascular unit must be rescued from ischemia [28].
2. Many of the potential targets have a biphasic cycle whereby the same mediator or molecule plays a different role under pathologic or physiological conditions. For instance, in the earliest phase of ischemic stroke the excitatory glutamate NMDA receptors become hyperactive and mediate cell death, but these same receptors are critical for neurogenesis and neuronal plasticity during the recovery phase of stroke. A similar mechanism occurs with metalloproteases [29,30,31,32,33,34] that contribute to the breakdown of the blood brain barrier (BBB) enlarging the ischemic lesion but are critical also for angiogenesis during the rec

Therefore, better animal models are required to explore the complexity of acute ischemic stroke. The use of preclinical STAIR criteria [35] provides adequate guidelines but even the strict adherence to these criteria does not predict clinical success.

Because of the above reasons, and despite the large number of neuroprotective agents that have been proposed to interrupt the ischemic cascade based on successful animal studies, most therapeutic clinical trials of these agents have yet to show consistent benefit. According to Sahota and Savitz [18] the most promising interventions that provide acute neuroprotection tested in larger clinical trials include hypothermia, magnesium sulfate, citicoline, and albumin. The most promising therapies enhancing neurorecovery in the subacute phase of stroke include granulocyte colony stimulating factor, G-CSF [36], citicoline, and cell-based therapies. Of all the above agents and methods, only citicoline appears to provide both neuroprotection and enhanced neurorepair with remarkable absence of side effects [37].

## 3. Citicoline Neuroprotection in Experimental Stroke

**Figure 1:**
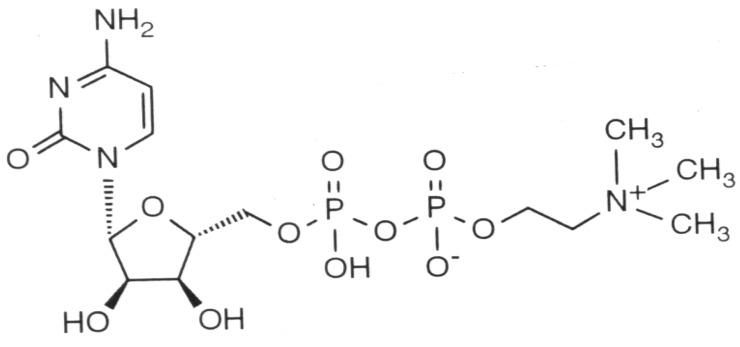
Chemical structure of citicoline, showing cytidine on the left and diphospho-choline (trimethyl-ethanol-ammonium) on the right. Cytidine is a nucleoside formed by cytosine attached to a ribose ring.

Citicoline (cytidine-5′-diphosphocholine or CDP-choline) is a naturally occurring endogenous compound, originally identified by Kennedy [38] in 1956 as the key intermediary in the biosynthesis of phosphatidylcholine. Citicoline is composed of two essential molecules, cytidine and choline (Figure 1), the structural phospholipids of cell membranes. Phospholipids are essential constituents of cells and have a high turnover rate, which requires the continuous synthesis of these compounds to ensure the adequate function of cell membranes. Damaged cell membranes and impaired metabolism of phospholipids have been implicated in the pathophysiology of cerebral ischemia. It appears that an important component of citicoline neuroprotective capacity is its ability to improve phosphatidylcholine synthesis in the injured brain [39,40].

A large number of research studies have explored the protective effects of citicoline in experimental stroke models [37,41]. At the experimental level, citicoline has been reported to decrease infarct volume and to reduce brain edema, with improvement of neurologic deficits either as a single therapy or in combination with other agents, including rtPA and nimodipine [42]. A large meta-analysis of experimental stroke studies with citicoline in ischemic stroke concluded that citicoline reduces infarct volume by 27.8% (19.9%–35.6% *p* < 0.001) [41]. However, as mentioned later, its effects vary with the dose whereby higher doses of citicoline produced greater reduction of brain damage compared with lower doses. Using a recent experimental model of stereotactic drug delivery to bypass the BBB delivering citicoline in direct contact with ischemic neurons in a MCA occlusion model in rats, Xu *et al*. [43] demonstrated optimal effects of citicoline administration by this stereotactic delivery method under MRI guidance.

Citicoline has therapeutic effects at several stages of the ischemic cascade in acute ischemic stroke. First, it stabilizes cell membranes by increasing phosphatidylcholine and sphingomyelin synthesis [37,44] and by inhibiting the release of free fatty acids [45]. By protecting membranes, citicoline inhibits glutamate release during ischemia. In an experimental model of ischemia in the rat, citicoline treatment decreased glutamate levels and stroke size [46]. Caspase is activated in human stroke [47] and citicoline has been shown to decrease the release of damaging caspase activation products [48] inhibiting apoptosis in animal models of brain ischemia [23]. Citicoline favors the synthesis of nucleic acids, proteins, acetylcholine and other neurotransmitters, and decreases free radical formation [49,50]. Therefore, citicoline simultaneously inhibits different steps of the ischemic cascade protecting the injured tissue against early and delayed mechanisms responsible for ischemic brain injury. Finally, citicoline may facilitate recovery by enhancing synaptic outgrowth and increased neuroplasticity [50] with decrease of neurologic deficits and improvement of behavioral performance, as well as learning and memory tasks [40].

## 4. Clinical Experience with Citicoline in Stroke Patients

For the past two decades, multiple randomized clinical stroke trials on citicoline reported the effectiveness of this pharmacological intervention when used early after onset of ischemia, as demonstrated by improvements in level of consciousness and modified Rankin score [51]. Given that various populations of stroke patients were included in these studies using different sample sizes, multiple doses, and several outcome endpoints, it became difficult to reach valid conclusions. Most studies, however, demonstrated a positive effect with the use of citicoline during the acute and subacute phases of ischemic stroke [52]. For instance, the *ECCO 2000* trial [53] included 90 patients that underwent diffusion-MRI prior to the onset of the treatment and a second one with T2 sequences 12 weeks later. Patients treated with 2 g daily of citicoline orally had an initial lesion volume of 62 mL and this was reduced six weeks later to 17 mL; in comparison with controls, the MRI reduction in infarct size was statistically significant [53]. Moreover, 70% of the patients with clinical improvement of greater than seven points in the NIH stroke scale had smaller stroke size compared with 42% in those without clinical improvement.

## 5. Data Pooling Analyses

In 2002, we performed a data pooling analysis to determine the effect of citicoline on neurological and functional recovery three months after moderate to severe stroke (baseline NIH ≥ 8) in comparison with placebo [54]. The main outcome measure was global improvement using Generalized Estimating Equations (GEE analysis), *i.e.*, the degree of neurological and functional recovery represented by the global scores of the NIH Stroke Scale (NIH-SS ≤ 1), Barthel’s Index (BI ≥ 95%) and the modified Rankin score (mRS ≤ 1). This study reviewed all randomized double-blind, parallel, placebo-controlled studies performed in patients with ischemic stroke treated with either citicoline or placebo within the first 24 h of the onset of symptoms and during a period of six weeks. The daily oral doses used ranged from 500 mg, 1000 mg, to 2000 mg. The patients included fulfilled the following criteria: age ≥ 18 years, randomized within the first 24 h after onset of stroke symptoms, persistent deficits for >60 min, brain CT and/or MRI compatible with the diagnosis of stroke, symptoms suggestive of acute ischemia in the MCA territory, baseline NIH score ≥ 8 (with at least two points from motor deficit), and mRS ≤ 1 prior to the stroke. Finally, subjects had none of the following exclusion criteria: brain CT/MRI with other structural lesions, serious systemic disease, unstable cardiovascular disease, pre-existing disability and/or psychiatric disease or dementia.

**Figure 2:**
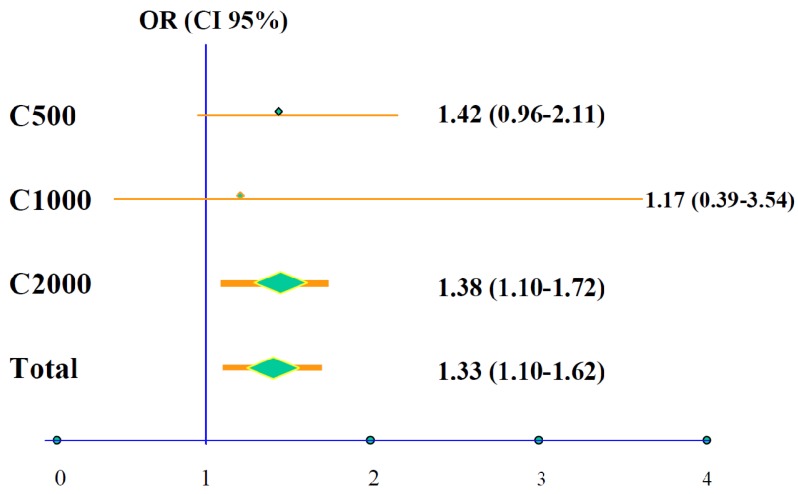
Probability of total recovery according to daily dose of citicoline among patients included in the pooled data analysis (*N* = 789 subjects on citicoline compared with 583 on placebo).

Following a comprehensive review, a total of 1372 patients were included in the data pooling analysis, 789 treated with citicoline and 583 with placebo, from four controlled clinical trials performed in the USA [55,56,57,58]. After 12 weeks of treatment 25.2% of the patients treated with citicoline presented complete recovery compared with only 20.2% of the placebo-treated cases (OR 1.33; 95% CI 1.10–1.62; *p* = 0.0034). As mentioned above, patients included in the data pooling analysis received three different daily doses of citicoline: 500 mg, 1000 mg or 2000 mg; the group treated with 2000 mg/day had statistically significant better prognosis with a 38% higher probability of complete recovery at 12 weeks (Figure 2) compared with those at lower doses.

Upon individual analysis of each one of the three variables that conform to the main global variable, it was determined that improvement occurred both with neurological deficits measured by the NIH-SS, as well as with functional scales (BI and mRS). In comparison with placebo-treated subjects, citicoline-treated patients reached a higher percentage of complete neurological and functional recovery. This was particularly clear with mRS scores (OR 1.42; 95% CI 1.08–1.88; *p* = 0.013). There were no differences in side effects or number of cases withdrawing from the trial between the two groups.

In summary, the results of the data pooling analysis concluded that patients with moderate to severe ischemic stroke (NIH ≥ 8) treated with citicoline orally within 24 h of onset for a period of six weeks demonstrated a statistically significant increase of 33% in the probability of achieving complete recovery at 12 weeks; furthermore, it was demonstrated that citicoline is a safe medication.

A meta-analysis by Sever [59] of 10 controlled clinical trials using citicoline studied 2279 patients, including both ischemic and hemorrhagic stroke distributed as follows, ischemic stroke: 1278 (1171 on citicoline *vs*. 892 controls) and 215 intracerebral hemorrhages (107 on citicoline *vs*. 109 controls). This meta-analysis demonstrated similar results to those of the data pooling analysis. In comparison with placebo, patients treated with citicoline showed significant reduction in the frequency of death or disability at follow-up (57.0% *vs*. 67.5%; OR 0.64; 95% CI 0.54–0.77; *p* < 0.001). Safety analysis showed no adverse effects in comparison with placebo (14.5% *vs*. 14.0%; OR 0.99; 95% CI 0.77–1.21; *p* = 0.94).

## 6. The ICTUS Trial

The International Citicoline Trial on Acute Stroke, ICTUS [60] was designed to confirm the encouraging results of the data pooling analyses and to replicate those trends. ICTUS was an international, multicenter, prospective, double-blind, randomized, placebo-controlled trial with participation of neurology services from 37 centers in Spain, 11 in Portugal, and 11 in Germany. Patients were randomized in a 1:1 ratio to citicoline or placebo. Citicoline was dosed at 2000 mg/day during six weeks; in the first three days it was given intravenously (1000 mg/12 h) and orally from the 4th day on for six weeks (two tablets 500 mg/12 h).

The main objective of the study was to confirm the results of the data pooling analysis; *i.e.*, to determine the overall effects of citicoline on moderate to severe ischemic stroke recovery (NIHSS at baseline ≥8) after three months of therapy with 2000 mg/day of citicoline (six weeks of treatment and 6 weeks of follow-up) in comparison with placebo. The global variable previously used in the data pooling analysis was the main end-point, with three components: neurological deficit (NIH-SS ≤ 1), functional capacity (mRS ≤ 1) and activities of daily living (BI ≥ 95). The main global variable was studied using GEE analysis.

The results were as follows [60]: from a total of 2298 patients enrolled into the study 1148 were assigned to citicoline and 1150 to placebo. The trial was stopped for futility at the 3rd interim analysis on the basis of complete data from 2078 patients. Global recovery at 90 days was similar in both groups. The median unbiased estimate of the adjusted odds ratio of the primary efficacy endpoint was 1.03 (95% CI 0.86–1.25). The odds ratios were also neutral in the sub-groups defined by minimization factors. Similar results were reported for each one of the secondary objectives (mRS ≤ 1, NIHSS ≤ 1, Barthel index ≥95). Mortality was comparable between the two groups (19% in the citicoline group *vs*. 21% in the placebo group). Adverse events occurred with similar frequency in both groups. Symptomatic hemorrhagic transformation occurred in 6% of patients who received citicoline and 8% of patients assigned to placebo (*p* = 0.25) [25].

The following are the main conclusions derived from the results of the ICTUS trial:

- Citicoline had no significant effect on the risk of hemorrhage from rtPA and had a comparable safety and tolerability profile compared to placebo.
- Global recovery at 90 days was similar in patients who received citicoline and in those who received placebo. Results were also neutral in the secondary endpoints and in the predetermined protocol analyses.
- Under the circumstances of the ICTUS trial, citicoline is safe but does not provide efficacy evidence for the treatment of moderate-to-severe acute ischemic stroke.

Some important characteristics of the ICTUS trial probably influenced the results:

1. Patients had more severe strokes in the ICTUS trial, as demonstrated by the NIH-SS 15 [11,12,13,14,15,16,17,18,19] *vs*. 14 [10,11,12,13,14,15,16,17,18] in previous studies; this renders more difficult the demonstration of a favorable effect; the main end-point required global improvement of both neurological and functional measurements. In fact, in the ICTUS trial the mRs 0–2 was 29% *vs*. 39% for pooled cases.
2. It is conceivable that larger doses for a longer period could have had a positive effect. In the previously noted meta-analysis of experimental data [41] greater reduction of infarct volume occurred in rats treated with larger doses of citicoline (300–500 mg/kg), along with superior recovery (27%; 95% CI 9–46) in comparison with animals treated with lower doses (100–300 mg/kg) with 18% recovery (95% CI 5–32; *p* > 0.001). Larger reduction of stroke volume was also documented in another study [61]; moreover, citicoline at high doses is as effective as *i.v*. thrombolysis in experimental stroke [62].
3. Patients enrolled in the ICTUS trial were not required to have neuroimaging studies of ischemic penumbra. Therefore, it was impossible to determine if at the onset of therapy salvageable brain tissue was present; moreover, this lack of images prevented accurate evaluation of stroke evolution. The latter is highly relevant given that in the *ECCO 2000 Citicoline Trial—DWI Sub-study* a comparison of DW-MRIs obtained at baseline with T2 MR images at week 12 of treatment with citicoline (2 g/day for six weeks) showed a significant decrease in volume of the cortical lesion [53]; this reduction in lesions size was associated with better clinical outcome, as mentioned above.
4. Finally, a substantial number of patients received *i.v*. rtPA rendering the analysis of the results more difficult since many patients reached the maximum possible recovery with the thrombolytic treatment. Thus, a *ceiling effect* resulting from an already maximal improvement due to rtPA effect cannot be ruled out. Almost half of the patients (47%) in the ICTUS trial received *i.v*. rtPA compared with only 13% in the pooled data analyses. Additionally, the trials were done 10 years apart, a period of time during which the standard of stroke care has improved substantially.

## 7. Hemorrhagic Stroke

A single clinical trial (*FI-CDPc-HIC*) has used citicoline in patients with hemorrhagic stroke [63]. This was a pilot, double-blind, randomized, placebo-controlled trial to evaluate the efficacy and safety of citicoline in patients with acute intracerebral hemorrhage (AICH). The study enrolled patients aged 40–85 years old with a primary hemispheric supratentorial hemorrhage within less than 6 h of evolution. Patients were treated with placebo or citicoline 1 g/12 h *i.v*. during the first week and then orally. Safety analysis showed no differences with placebo in terms of adverse effects, mortality or study withdrawals. The results showed that 6.7% of the patients treated with placebo had reached independence (Rankin 0–2) at 12 weeks compared with 27.8% of those on citicoline. In conclusion, citicoline is a safe and effective pharmacological product in patients with AICH and can be used in acute stroke patients even before images are obtained to separate ischemic from hemorrhagic stroke.

## 8. Brain Neurorepair

Spontaneous recovery of function occurs naturally after stroke in both humans and in animal models. This functional recovery is generally incomplete and results from reversal of diaschisis, activation of cellular genesis, repair mechanisms, change in the properties of the existing neuronal pathways and stimulation of neuronal plasticity leading to new neuronal connections [64,65].

In patients with ischemic stroke neurological recovery occurs over a period of three months, and this is the usual evaluation time for final outcome in neuroprotection trials. However, recovery is only possible when neurorepair occurs, including not only repair of the damaged neurons, but also enhancement of angiogenesis [66] and brain plasticity (neuronal and synaptic).

The adult human brain has the capacity to undergo physiological and anatomical modifications leading to motor and cognitive recovery [67]. Cerebral ischemia launches concurrently neurogenesis and angiogenesis, two closely interconnected processes that enhance neural repair.

There is definitive evidence that neurogenesis occurs in the adult brain following a stroke. Endogenous progenitor neural stem cells are normally present in the normal brain and maintain the capacity to produce new neurons and glial cells during adult life. Progenitor neural stem cells capable of producing neuroblasts in the adult human brain are situated in the subventricular zone of the lateral ventricle and in the dentate gyrus of the hippocampus. Under physiological conditions the neuroblasts of the subventricular zone migrate towards the olfactory bulb where they are transformed into neurons. In response to brain ischemia, the adult progenitor neural cells proliferate in the ipsilateral subventricular zone and migrate towards the zone surrounding the infarction where they mature into adult neurons that may become part of functional neuronal circuits [68].

Neuropathological studies have shown the increase in cellular proliferation and in neuroblasts in the subventricular zone in patients who died shortly after an acute ischemic stroke [68]. However, many of the newly formed immature neurons and neural cells die and are never integrated into functional neuronal circuits. For this reason, it is important to develop novel cellular and pharmacological strategies to increase neurogenesis leading to functional neuronal circuits. Repair of focal cortical strokes [69] is not done by neuroblasts migrating from the subventricular zone but from clonal neural spheres originating from the peri-infarct area that differentiate into neurons, astrocytes, oligodendrocytes, and smooth muscle cells.

Angiogenesis [66] is one of the main components of the processes of post-ictal neurovascular remodeling. It induces capillary neoformation in response to proliferation and migration of primordial stem cells originating from the existing blood vessels. The pericytes appear to have a major role in neurogeneration responses. The pericyte is a pluripotent stem cell in the brain with the potential of differentiating into cells of neural lineage such as astrocytes, oligodendrocytes and neurons [70]. Angiogenesis can be observed several days following an ischemic stroke and it has been shown that a higher capillary density correlates with longer survival. Proangiogeneic factors such as vascular endothelial growth factor or VEGF [71], and metalloproteinases increase following cerebral ischemia. The effect of angiogenesis is to increase collateral circulation to meet the metabolic demands in terms of oxygen, glucose and nutrients required by the damaged and repaired tissues. Also, the newly generated blood vessels provide the neurotrophic support required by neurogenesis and synaptogenesis that eventually lead to functional recovery. In summary, angiogenesis provides the stimulation required to launch and enhance endogenous mechanisms repair and recovery including neurogenesis and synaptogenesis, as well as neuronal and synaptic plasticity. These events are all involved in the long-term repair and restoration process that take place in the brain after acute or chronic ischemic events [72]; therefore, angiogenesis is one of the most promising areas of research in the field of stroke treatment [66,67].

## 9. Neurorepair Therapies

Repair therapies aim to restore the brain, a goal that differs from that of neuroprotection therapies, in which the aim is to limit acute stroke injury. A number of potentially useful post-stroke interventions are currently being evaluated, such as the “mirror therapy” [73] that is simple and useful to apply in addition to traditional physical therapy and rehabilitation treatments. Neuromuscular electrical stimulation has been found to improve neuromuscular function and to stimulate cerebral plasticity [74].

Transcranial magnetic stimulation [75], in addition to physical and occupational therapy, significantly improves motor function. Improvement is due to stronger stimulation of intact motor cortical regions homolateral to the hemiplegic side [75].

The NEST-3 (NeuroThera^®^ Efficacy and Safety Trial-3) trial [75] is currently being conducted. This is a multicenter, double-blind, randomized, placebo-controlled pilot study with parallel groups to evaluate the safety and efficacy of a transcraneal laser stimulation with the NeuroThera^®^ Laser System in patients within 24 h of an acute ischemic ictus. Finally, there is an enormous potential with the use of robotic therapy after stroke [75].

A number of medications have been used to enhance recovery and tissue repair following ischemic stroke. Among the anti-depressants, serotonine uptake inhibitors (SSRIs) and noradrenergic inhibitors have been demonstrated to improve motor recovery in patients with ischemic stroke [76,77]. The mechanism of action of SSRIs is unknown. Acler and colleagues [78] described decreased excitability of the threshold of the contralateral motor cortex after one month of use of citalopram. Decreased contralateral threshold increases motor recovery; neurogenesis and synaptic plasticity when the treatment is used for periods as long as one year. Valproic acid treatment appears to decrease stroke size in experimental stroke in rats, probably by enhancing angiogenesis in the hemisphere ipsilateral to the arterial occlusion [79].

## 10. Citicoline and Brain Neurorepair

In addition to the neuroprotective effects, citicoline also possesses a substantial neuroregenerative potential that may explain better its long-term beneficial effects in post-stroke patients.

In an experimental stroke model with permanent occlusion of the distal MCA in mice citicoline (500 mg/kg) or vehicle was administered 24 h later intraperitoneally for 1–2 weeks. Citicoline treatment decreased neuronal apoptosis and promoted endogenous cerebral repair [80]. A well-known experimental study conducted at Madrid’s Complutense University demonstrated that treatment with citicoline 24 h after MCA occlusion in rats produced an increase in neuronal synaptic spines with increased motor and functional recovery in treated animals [50].

Endothelial progenitor cells (EPCs) are circulating immature pluripotential hematopoietic cells capable of differentiating into mature endothelial cells to help in the recovery of capillary and vascular recovery of ischemic areas. EPCs also promote growth factor release and increase neurogenesis. The increase in circulating EPCs after acute ischemic stroke is associated with good functional outcome, reduced infarct growth and neurological improvement. It has been shown that increase in EPCs in peripheral blood in acute stroke patients improves functional recovery and decrease stroke size [81]. In a prospective study including 48 patients with a first-ever non-lacunar stroke citicoline treatment and the co-treatment with citicoline and rt-PA are independently associated with a higher increase in circulating EPCs during the first week in acute ischemic stroke [82]. Gutiérrez-Fernández *et al*. [83] demonstrated in an experimental model of stroke in rats that treatment with CDP-choline significantly improved functional recovery associated with a decrease in lesion volume by MRI, less cell death and decreased expression of low-density lipoprotein receptor-related protein (LRP). In fact, CDP-choline increased cell proliferation, vasculogenesis and synaptophysin levels and reduced glial fibrillary acidic protein (GFAP) levels in the peri-infarct area of the ischemic stroke. A more recent study on 40 rats treated at 24 h of experimental stroke with citicoline during 10 days showed significant improvement in both motor and somatosensory recovery by increasing neurogenesis in the peri-infarct area, subventricular zone and dentate gyrus [84].

**Figure 3:**
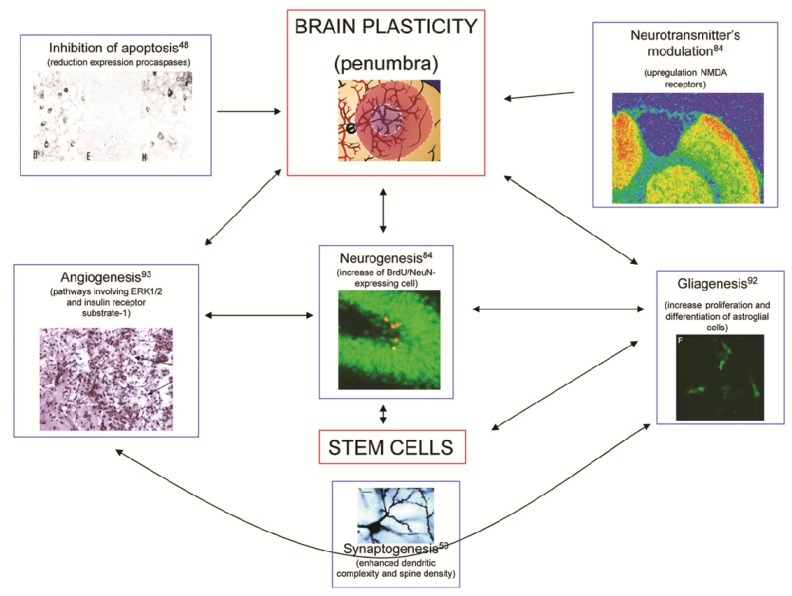
Major mechanisms involved in brain plasticity. The diagram explains the actions of citicoline to enhance the processes of inhibition of apoptosis [48], angiogenesis [92], neurogenesis [84], gliagenesis [93], synaptogenesis [50], and modulation of neurotransmitters [84]. Notice that all these effects are similar to those induced by stem cells.

In summary, citicoline enhances both brain neuroprotective and neurorepair mechanisms following ischemic stroke. These mechanisms are illustrated in Figure 3.

## 11. Citicoline in Post-Stroke Cognitive Decline

Cognitive and behavioral manifestations are frequently observed in patients with vascular cognitive impairment and vascular dementia. Cognitive impairments occur in nearly half of stroke survivors [85], a frequency more elevated than that of stroke recurrence. These impairments may be more important determinants of functional outcomes after stroke than physical disability [86,87].

**Figure 4:**
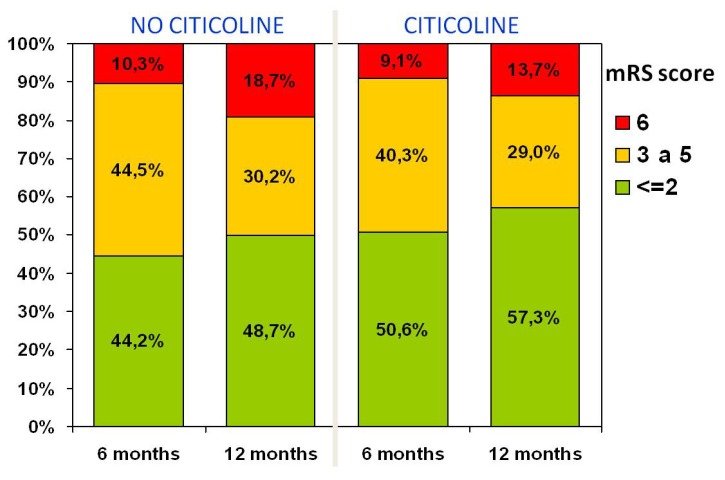
Functional status during follow-up: Notice the improvement in mRS scores (<2) at six and 12 months following stroke in the group treated with citicoline, compared with those untreated. From Álvarez-Sabín *et al*. [91].

**Figure 5:**
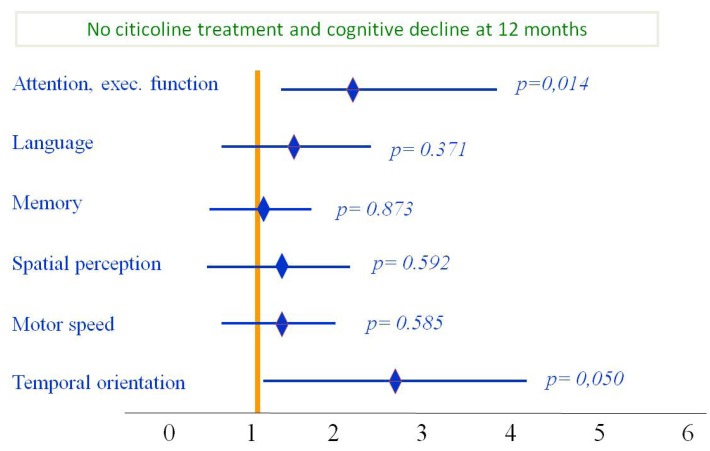
Functional status during six month follow-up: Subjects treated with citicoline had improvement on all cognitive domains; however, improvement was statistically significant only for attention/executive function and temporal orientation. Modified from Álvarez-Sabín *et al*. [91].

Most end-points used in clinical trials address issues relevant to motor function, activities of daily living and quality of life; in fact, many patients with cognitive or behavioral problems are excluded from clinical trials. Therefore, there is a need to identify cognitive and behavioral problems occurring as a result of stroke or “silent” small-vessel vascular disease. For the above reasons, International Guidelines recommend routine cognitive and behavioral evaluation of stroke patients [88]. In reality, these aspects are rarely evaluated in stroke patients [89]. Along the same lines, few pharmacological products have been evaluated for prevention or treatment of cognitive problems in the stroke patient. A Cochrane meta-analysis of citicoline in 942 patients with vascular cognitive impairment studied in 12 placebo-controlled, double-blind, randomized studies showed modest evidence of improvement in memory and behavior, and a significant impression of improvement on the global impression of change on the part of caregivers [90]. Based on these data and on abundant evidence on the neuroprotective and neurorepair effects of citicoline, we evaluated the safety and efficacy of citicoline on the cognitive manifestations of patients with acute ischemic stroke. This study was an open-label, randomized, parallel study of citicoline (1 g/day) for 12 months *vs*. usual treatment in patients with first-ever ischemic stroke [91]. Citicoline-treated patients showed better outcome at follow-up in attention-executive functions and temporal orientation at six months and 12 months (Figure 4). Moreover, although differences are not statistically different, patients treated with citicoline showed a trend towards having a better functional outcome, measured with mRS at 6 and 12 months (Figure 5).

## 12. Expert Opinion

In conclusion, citicoline, a naturally occurring endogenous compound, is a key intermediary in the biosynthesis of phosphatidylcholine. Long-term treatment with citicoline is remarkably safe and has demonstrated therapeutic effects at several stages of the ischemic cascade in acute ischemic stroke with demonstrated efficacy in numerous animal models of acute stroke. Long-term treatment with citicoline is safe and effective, improving post-stroke cognitive decline and enhancing patients’ functional recovery. Prolonged citicoline administration at optimal doses has been demonstrated to be remarkably well tolerated and to enhance endogenous mechanisms of neurogenesis and neurorepair, similar to those obtained with stem cells [92,93], contributing to physical therapy and rehabilitation.
